# Ophthalmic findings in patients with autosomal recessive lamellar ichthyosis due to *TGM1* mutations in an isolated population

**DOI:** 10.1007/s10792-023-02774-3

**Published:** 2023-08-05

**Authors:** Nicole Macriz-Romero, Guillermo Raul Vera-Duarte, Jesus Guerrero-Becerril, Oscar Francisco Chacón-Camacho, Mirena C Astiazarán, Juan Carlos Zenteno, Enrique O. Graue-Hernandez

**Affiliations:** 1 Department of Cornea, External Disease and Refractive Surgery, Institute of Ophthalmology “Conde de Valenciana”, Chimalpopoca #14, Colonia Obrera, Cuauhtémoc, 06800 Mexico City, Mexico; 2 Department of Genetics, Institute of Ophthalmology “Conde de Valenciana”, Mexico City, Mexico; 3 Iztacala Faculty of Superior Studies, National Autonomous University of Mexico (UNAM), Mexico City, Mexico; 4 Biochemistry Department, Faculty Medicine, National Autonomous University of Mexico, Mexico City, Mexico; 5 Rare Diseases Diagnostic Unit, Faculty of Medicine, National Autonomous University of Mexico (UNAM), Mexico City, Mexico

**Keywords:** Lamellar ichthyosis, Ichthyosis, Ectropion, TGM1

## Abstract

**Purpose:**

To describe the ocular clinical characteristics of a group of Mexican patients with lamellar ichthyosis (LI) arising from TGM1 pathogenic variants.

**Methods:**

Ophthalmological exploration, pedigree analysis and genetic screening were performed in patients with an established clinical diagnosis of lamellar ichthyosis from families located in a small community in the Southeast of Mexico.

**Results:**

Nine patients with LI in five families were identified. There were six affected females. All patients (9/9) demonstrated eye lid abnormalities with eight patients showing lid margin abnormalities. Madarosis was present in only three individuals and corneal scarring was documented in two. All nine individuals carried biallelic TGM1 variants, either homozygously or as compound heterozygous.

**Conclusion:**

Ocular anomalies are common in individuals with TGM1-related LI. The occurrence of a variety of private or rare mutations hampers the identification of a genotype–phenotype correlation for ocular anomalies in this disorder.

## Introduction

The term “ichthyosis” derives from the Greek word “*ichthys,*" meaning fish, and it has been used for over 200 years to describe a group of diseases characterized by generalized desquamation of the skin, generalized “scales,” dry skin, hyperkeratosis, and sometimes erythroderma [1, 2]. Ichthyoses can occur as an acquired or hereditary trait, with a congenital or late onset presentation and it can appear as an isolated entity or in association with other anomalies. While some forms of ichthyosis are clinically well defined and can be relatively easily to diagnose, precise diagnosis can be challenging to stablish due to their significant clinical variability. Hereditary ichthyosis is a Mendelian etiologically heterogeneous disease classified in two large groups: non-syndromic forms that manifest primarily in the skin, and syndromic forms, with extracutaneous associated anomalies [3]. The non-syndromic form comprises four entities: ordinary ichthyosis, autosomal recessive congenital ichthyosis (ARCI), keratopathic ichthyosis, and other less frequent forms of ichthyosis.

ARCI has an estimated prevalence of 1/200,000 individuals in the United States of America. Harlequin ichthyosis is the rarest form, and it presents in 1/1,000,000 births [4, 5]. Epidemiological studies in Norway and the Spanish community of Galicia have identified founder mutations responsible for the particularly high prevalence of ARCI in those populations, with 1/91,000 and 1/122,000, respectively [6, 7]. ARCI clinical manifestations include neonatal dehydration, ectropion, recurrent skin infections, hypohidrosis with severe intolerance to heat and eclabium. To date, there are nine known genes associated with ARCI although no clear genotype–phenotype relationship and different mutations in the same gene can present with different phenotypes [4].

Lamellar ichthyosis (LI), one of the least prevalent and most severe forms of congenital ichthyosis, is characterized by thick and gray or brown squama that covers all over body present at birth and persisting for life. The most commonly affected genes in LI are *TGM1*, *ALOXE3*, *NIPAL4*, *CYP4F22* and *ALOX12B*. *TGM1* mutations underlie 74–85% of LI cases [7–9].

Ocular manifestations of LI include exposure keratitis secondary to ectropion, unilateral megalocornea, enlarged corneal nerves, blepharitis, the absence of Meibomian glands, trichiasis, madarosis, and the absence of lacrimal puncta [10]. Al-Amry described ectropion of both the upper and lower eyelids and no conjunctival involvement as the most common ocular findings in a case series; they found ocular complications were not severe in most cases, according to previous reports [11]. Ocular surface changes include mild to moderate exposure keratopathy, secondary to lid abnormalities, trichiasis, and the absence of lacrimal punctum. Ectropion, however, does not improve spontaneously, and it tends to cause lagophthalmos with secondary corneal exposure, corneal ulceration, and in severe cases, perforation and phthisis bulbi [12]. Patients suffer from eye disease throughout life due to Meibomian gland dysfunction [13]. In this work, the ocular clinical characteristics in a series of Mexican patients with lamellar ichthyosis due to *TGM1* mutations are described.

## Methods

Ophthalmological evaluation was performed in subjects with established clinical diagnosis of lamellar ichthyosis. All participants gave written informed consent prior to inclusion in the study. Examination was performed by a single ophthalmologist and included best-corrected visual acuity determination, slit-lamp biomicroscopy, fundoscopy, and applanation tonometry. A geneticist investigated systemic anomalies. Family history and pedigrees were collected as well as oral mucosa cells for genomic DNA isolation (Gentra Puregene Buccal Cell—Qiagen, Hilden, Germany) and subsequent mutational screening of the TGM1 gene by PCR amplification direct Sanger sequencing using the program PrimerQuest® IDT, Coralville, U.S.A.). All procedures were performed in the Genetics Laboratory of the Research Unit at the Institute of Ophthalmology “Conde de Valenciana” in Mexico City. The study was performed under adherence of the ethical foundations of the Declaration of Helsinki and approved by the Ethics Commission of the Institute of Ophthalmology “Conde de Valenciana.”

## Results

**Fig. 1:**
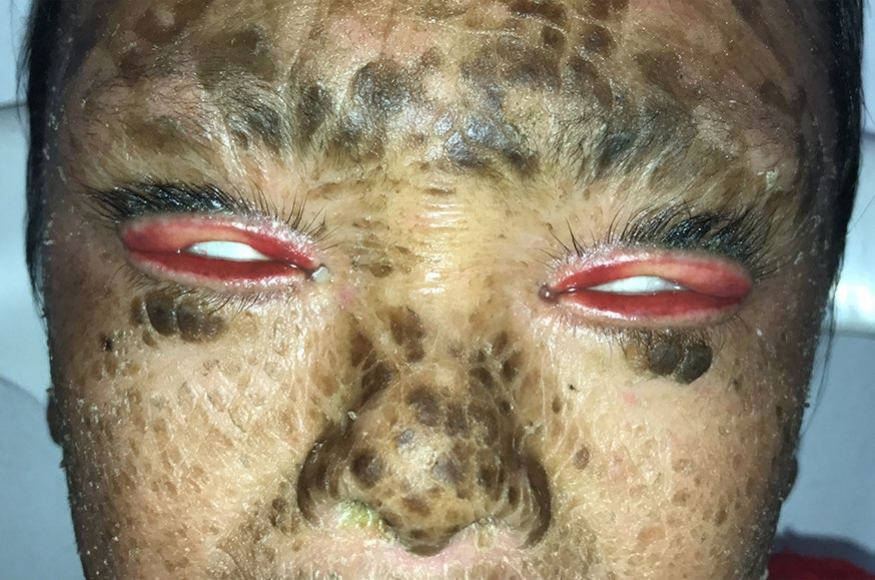
Patient 2 demonstrates spontaneous upper lid eversion in both eyes with eyelid closure

**Fig. 2:**
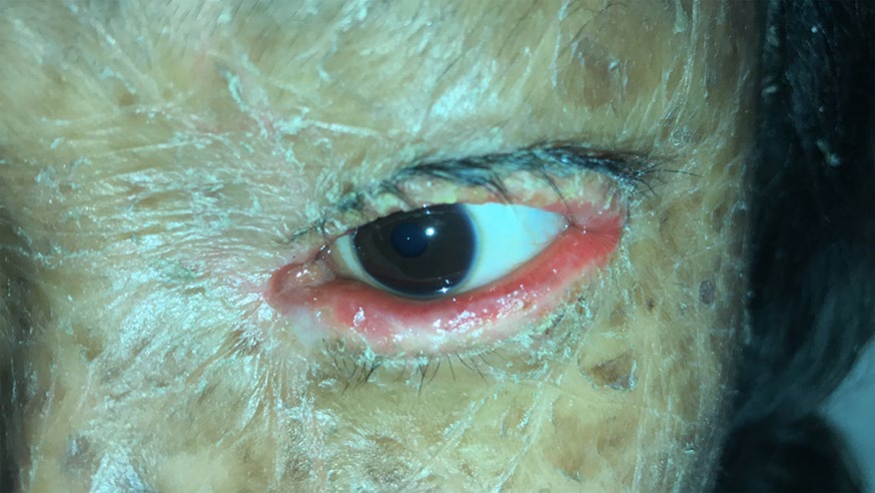
Patient 6 exhibits lid margin keratinization, lower lid madarosis and ectropion

**Fig. 3:**
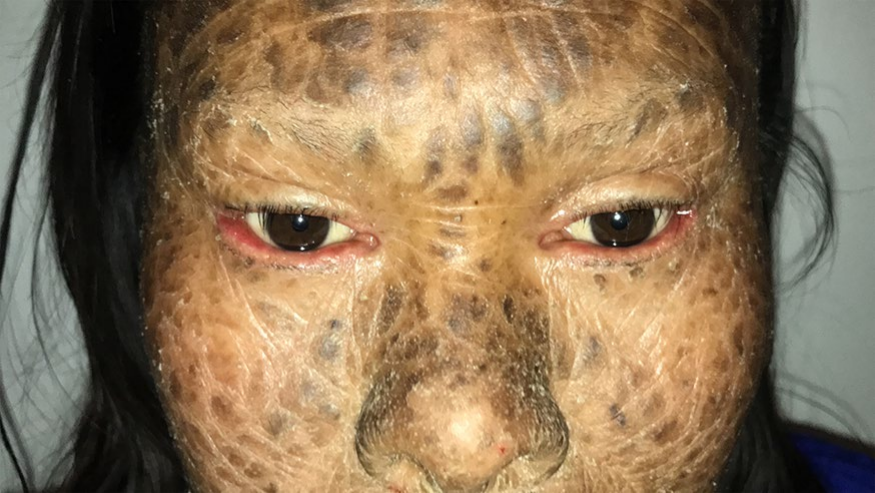
Patient 5 exhibits lid margin keratinization and ectropion

**Fig. 4:**
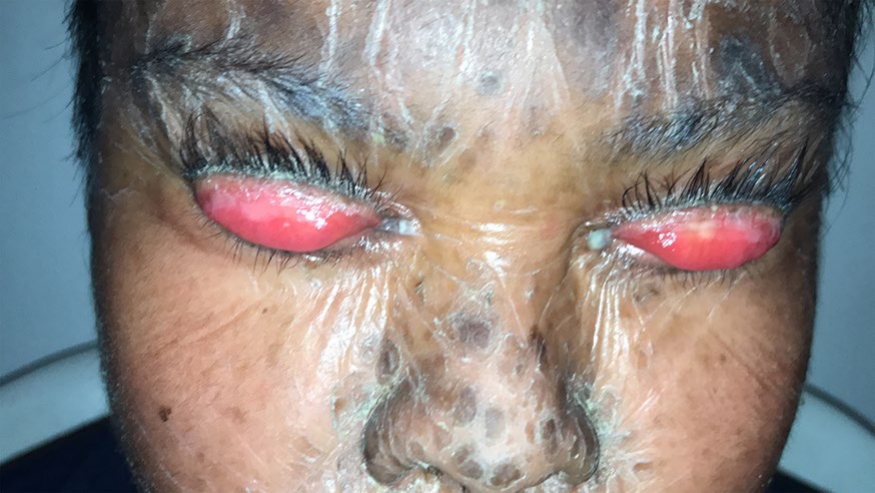
Patient 9 exhibits lid margin keratinization, lid madarosis and euryblepharon

**Fig. 5:**
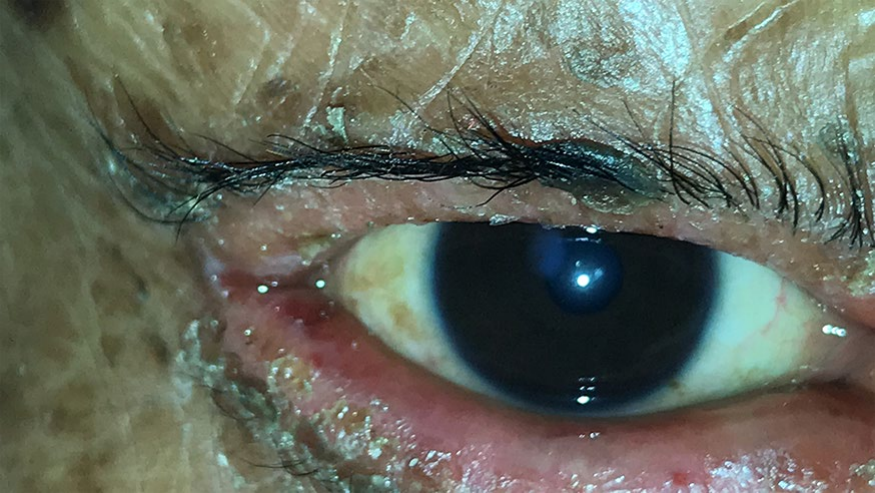
Patient 4 demonstrates corneal opacity secundary to the lid pathology

**Fig. 6:**
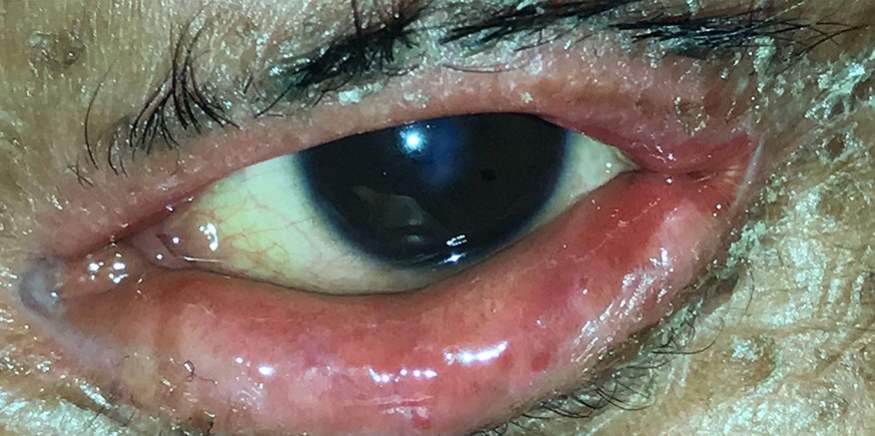
Patient 4 demonstrates lacrimal punctum keratinization, corneal scarring, and exposure keratopathy

Nine patients from five LI families were identified. The subjects pertained to 5 families settled in a small community of the Veracruz state (south Mexico). All of the patients (9/9) presented with the classic LI phenotype of dark brown scales distributed throughout the body. LI was present in all of them at birth, and none presented atypical features of the disease. The genealogical tree analysis supported autosomal recessive transmission in all families. Six out of nine patients (67%) were females. The youngest patient was 1 year old, while the oldest one was 27 years old. All patients (9/9) demonstrated palpebral abnormalities (Figs. 1, 2, 3, 4, 5 and 6). Seven (77%) presented ectropion, while lagophthalmos was present in four patients (44%). Eyelid shortening was exhibited in four patients (44%). Euryblepharon was observed in two patients (22%). Unilateral spontaneous eversion was present in two patients (22%), one on the left inferior eyelid and the other on the upper right eyelid. Shortening of the anterior lamella was present in two patients (22%). Eight patients (88%) showed lid margin abnormalities, being the most common lid margin keratinization (5/9, 55%), followed by lacrimal punctum abnormalities as a group (4/9, 44%). Of these, two patients (22%) demonstrated lacrimal punctum keratinization in both eyes: One patient showed inferior lacrimal punctum obstruction in one eye and another patient exhibited lacrimal punctum stenosis in both eyes (Figs. 5 and 6). Three patients (33%) presented with eyelash abnormalities, two with inferior madarosis in both eyes, while the third presented inferior madarosis only in the right eye. One of the patients as mentioned above also displayed unilateral superior trichiasis. Two patients (22%) displayed Meibomian gland dysfunction and one patient exhibited entropion. Regarding conjunctival abnormalities, four patients (44%) revealed a papillary reaction in both eyes. The cornea was affected in two patients (22%), one presenting a central corneal opacity in the right eye and a paracentral nasal opacity in the left eye. In contrast, the other patient displayed a central opacity only in the left eye. Clinical data are summarized in Table 1.

*TGM1* genetic analysis identified that seven subjects (66%) were homozygous for the c.427C > T (p.Arg143Cys) variant in exon 3, one patient was homozygous for the c.760G > A (p.Asp254Asn) variant in exon 5, and one subject was compound heterozygous for the c.427C > T/c.760G > A variants. This was an unexpected finding as all patients originated from the same small community and homozygosity for a single variant was assumed a priori. A summary of the clinical characteristics and the pathogenic *TGM1* variants in all nine patients is shown in Table 1.

**Table 1:** Clinical characteristics and TGM1 variant summarized

Patient #	Age (years)	Gender	Visual acuity	Eyelash anomalies	Palpebral anomalies	Lid margin anomalies	Conjunctival anomalies	Corneal anomalies	*TGM1* variant (zygosity)
1	1	F	Fixate and follow	–	Ectropion OU, lagophthalmos OU, eyelid shortening OU	MGD OU	–	–	c.427C > T (Homozygous)
2	6	M	20/20 OU	–	Spontaneous upper eversion OU, inferior ectropion OU, eyelid shortening OU	–	Papillary reaction OU	–	c.427C > T (Homozygous)
3	27	F	20/20 OU	Inferior madarosis OU	Ectropion OU, superior lid shortening OS	Lacrimal punctum keratinization OU	–	–	c.427C > T (Homozygous)
4	16	M	20/40 OU	–	Lagophthalmos OU, lid shortening OU	Lacrimal punctum keratinization OU	–	Central opacity OD, paracentral nasal opacity OS	c.760G > A (Homozygous)
5	17	F	20/20 OU	Superior trichiasis OD, inferior madarosis OU	Ectropion OU, euryblepharon OU	Lid margin keratinization OU, inferior lacrimal punctum obstruction OS	Papillary reaction OU	–	c.427C > T (Homozygous)
6	5	F	20/40 OU	Inferior madarosis OD	Ectropion OU, anterior lamella shortening OD, Lagophthalmos OD	Lid margin keratinization OU	–	–	c.427C > T (Homozygous)
7	5	F	20/40 OU	–	Ectropion OU, lagophthalmos OU	Lid margin keratinization OU, MGD OU	–	–	c.427C > T/ c.760G > A (Compound heterozygous)
8	17	F	20/80 OU	–	Spontaneous inferior eyelid eversion OS	Lid margin keratinization OU, lacrimal punctum stenosis OU	Papillary reaction OU	Central opacity OS	c.427C > T (Homozygous)
9	11	M	20/60 OU	–	Ectropion OU, anterior lamella shortening OU, euryblepharon OD, superior entropion OD	Lid margin keratinization OU	Papillary reaction OU	–	c.427C > T (Homozygous)

*OU, oculus uterque (both eyes), *OD*, oculus dexter (right eye), *OS*, oculus sinister (left eye)

## Discussion

LI is the rarest and the most severe form of autosomal recessive ichthyoses. Individuals are born as collodion babies, with the membrane gradually exfoliating a few weeks after birth, only to be replaced by a scaling rash. The rash is generalized with skin flexures, palms, and soles affected [14]. Ocular anomalies are frequent in LI patients although a genotype–phenotype correlation has not been established yet.

As reported in the literature, ectropion of both the upper and lower eyelids is the most common finding in LI patients, with an estimated frequency between 45 and 80% [11]. Arnold was the first to report the association in 1834, and it occurs only in the lamellar type of ichthyosis [14]. The ectropion of LI is cicatricial in nature and appears to be a result of excessive dryness of the skin and subsequent contracture [15]. It is frequently bilateral and the lower lid is more severely affected. In the present group of patients, ectropion was observed in 7/9 patients and always in a bilateral fashion. Singh et al. reported a LI case with entropion, an unusual finding in the disease since lash ptosis usually occurs in conditions where the anterior lamella is loose [14]. Interestingly, one of our patients exhibited superior entropion of the right eye. The mechanism for this anomaly is ostensibly the same as that which causes ectropion, i.e., chronic inflammation in the anterior lamella of the eyelid skin [14]. A characteristic of ichthyosis is trans-epidermal dehydration and loss of elasticity and contraction of palpebral skin; thus, vertical shortening is seen in the anterior lamella with consequent ectropion arises [16, 17].

Conjunctival anomalies develop only in the autosomal recessive type of ichthyosis [18]. Buller et al. reported conjunctival changes, describing the conjunctiva of the lower lids as mildly swollen, having a smooth rather than glazed appearance, and presenting several longitudinal ridges without any follicular reaction [19] . Katowitz et al. reported keratinization and papillary formation of the conjunctiva [20] and Singh et al. reported no conjunctival involvement with LI [14]. In the present series, four patients (44%) exhibited a tarsal papillary reaction.

Ocular surface complications in LI are usually attributed to cornea exposure secondary to ectropion and lagophthalmos [14, 15]. In our series, two patients (22%) exhibited corneal opacities. One of them had bilateral opacities in eyes that were also affected by lagophthalmos and lid shortening and therefore can be attributed to exposure keratopathy. The other patient displayed a corneal opacity in only one eye that showed spontaneous inferior eyelid eversion. The absence of corneal opacities in the rest of the patients can be explained Bell´s phenomena. None of the patients demonstrated severe ocular complications, as has previously been reported by several authors [11]. Further clinical examination is still needed to elucidate the full spectrum of ocular surface alterations in LI. Of particular interest would be meibography and tear film analysis, epithelial mapping, and impression cytology of the corneal and conjunctival epithelium. Like the skin, meibomian glands are of ectodermal origin and are likely affected in LI [13]. Meibomian gland dysfunction can be presumed to be present in all patients. These patients benefit from the constant use of an appropriate ocular lubricant and lifestyle modifications. Lipid-containing lubricant eye drops to restore the balance of the tear film are largely recommended [13].

The management and treatment of LI patients must be multidisciplinary and according to the severity with topical and/or oral agents. It involves the use of hydrating and lubricating agents, keratolytics (e.g., salicylic acid, urea, and lactic acid), and modulators of keratinocyte differentiation (e.g., retinoic acid) [16].

In general management of lid malposition with exposure keratopathy requires surgical intervention involving the use of skin grafts, often full layer grafts [21]. If the exposure keratopathy is severe the use of soft bandage contact lenses, amniotic membrane grafts, and tarsorrhaphy should also be considered in order to prevent potentially sight threatening complications such as corneal perforation [11].

Our patients were all treated judiciously as none of them presented with severe ophthalmic disease. Artificial tears and lifestyle modifications including daily bathing with water or mild cleanser and the application of plain emollients directly after bathing, as well as frequently throughout the day, help to seal in moisture [16]. Theoretically bathing aids to hydrate and promote shedding of the stratum corneum, therefore reducing the thickness of scaling and improving overall skin. They were all prescribed moisturizing cream with 7.5% urea that is both keratolytic and moisturizer [22].

## Conclusion

Finally, biallelic *TGM1* mutations were demonstrated in all nine patients, as expected for an autosomal recessive trait as LI. However, an unanticipated finding was the occurrence of 3 distinct TGM1 genotypes: 7 homozygous for c.427C > T(p.Arg143Cys), 1 homozygous for c.760G > A (p.Asp254Asn), and 1 compound heterozygous c.427C > T/c.760G > A. Both the c.427C > T and the c.760G > A variants have been previously published in LI patients [23, 24]. The identification of more than one pathogenic allele in “closed” populations has been described in several recessive diseases and has been attributed to the occurrence of sporadic waves of immigration with introduction of additional founders [25]. However, additional studies are required to confirm this possibility in the LI population described here.
